# Potential Psychological and Biological Mechanisms Underlying the Effectiveness of Neonatal Music Therapy during Kangaroo Mother Care for Preterm Infants and Their Parents

**DOI:** 10.3390/ijerph18168557

**Published:** 2021-08-13

**Authors:** Łucja Bieleninik, Mark Ettenberger, Shulamit Epstein, Cochavit Elefant, Shmuel Arnon

**Affiliations:** 1 Department of Clinical and Health Psychology, Faculty of Social Sciences, Institute of Psychology, University of Gdansk, 80-309 Gdansk, Poland; 2 GAMUT—The Grieg Academy Music Therapy Research Centre, NORCE Norwegian Research Centre AS, 5029 Bergen, Norway; 3 Music Therapy Service, University Hospital Fundación Santa Fe de Bogotá, Bogotá 110111, Colombia; mark.ettenberger@gmx.at; 4 SONO—Centro de Musicoterapia, Bogotá 110221, Colombia; 5 School for Creative Arts Therapies, University of Haifa, Haifa 3498838, Israel; shushuq@gmail.com (S.E.); celefant@univ.haifa.ac.il (C.E.); 6 Department of Neonatology, Meir Medical Center, Kfar Saba 44281, Israel; shmuelar@clalit.org.il; 7 Sackler Faculty of Medicine, Tel Aviv University, Tel Aviv 69978, Israel

**Keywords:** music therapy, MT, kangaroo care, NICU, premature infants, mechanism, pituitary–adrenal axis, autonomic nervous system, bonding

## Abstract

Neonatal music therapy (MT) has become more accessible worldwide. Previous research suggests multiple benefits of MT for preterm infants and their caregivers; however, far too little attention has been paid to understanding the mechanisms of change in previous Neonatal Intensive Care Unit (NICU)-MT research so far. This perspective article describes potential mechanisms of MT interventions exposed during kangaroo mother care on the preterm infant’s response (behavioral and physiological outcomes) and the mother-infant relationship. The paper focuses on the hypothalamic–pituitary–adrenal axis’ role in stabilization of behavioral state, the autonomic nervous system’s role in stabilization of physiologic state, as well as co-regulation as a potential mechanism for the developing of the parent-infant relationship. Mechanisms play a pivotal role in understanding variables related to the therapy course and well as in generating new knowledge regarding treatment susceptibility and optimizing resources. Understanding of the mechanisms of how interventions may lead to specific outcomes plays an important role in addressing the issue of improvement of currently available approaches of MT used in the NICU.

## 1. Introduction

The evidence-based movement within music therapy (MT) research in the Neonatal Intensive Care Unit (NICU) has been constantly growing over the last several decades, providing empirical support for the effectiveness of MT on treatment success for both preterm infants and their parents. NICU-MT is a non-pharmacological, non-invasive, and empirically supported treatment which in recent years has been increasingly included as part of standard care in many countries [1]. Systematic reviews [2,3,4,5,6,7] and meta-analyses of randomized controlled trials (RCTs) [8,9,10,11] have indicated that MT and music-based interventions bring benefits for both preterm infants and their caregivers. With respect to the latter reviews, Bieleninik, Ghetti, and Gold [8] conducted a meta-analysis of 14 RCTs involving 964 preterm-born infants and 266 parents and found a significant large effect of MT for infant respiratory rate (RR) and maternal anxiety. Yue and colleagues [11] included 13 RCTs with 1093 preterm-born infants and reported a favorable effect of MT on heart rate (HR), respiratory rate (RR), oral feeding volume, stress level, and maternal anxiety. Standley [9] concluded that MT is beneficial for infants with birth weight below 1000 g, postmenstrual age of <28 weeks, and when music is performed live and used early in the infant’s NICU stay. Van der Heijden and colleagues [7] reviewed 20 RCTs including 1128 participants and revealed that live MT and the use of recorded music can improve sleep, HR, as well as outcomes related to feeding skills. The systematic review of nine RCTs performed by Hartling and colleagues [12] showed benefits of music for physiologic parameters, behavioral states, oral feeding rates, and pain among preterm infants. However, little knowledge is available about the potential mechanisms in producing such clinical change.

According to Moldovan and Pintea [13] “a mechanism of change refers to the process or series of events through which one variable leads to and/or causes change in another variable. Mechanisms of change reflect the processes through which some independent variable (i.e., therapy) actually produces the change and explain how the intervention eventually leads to the outcome” (p. 301). Lerner and colleagues [14] define a mechanism as “a process or specific therapeutic ‘ingredient’ that can be said to be reliably responsible–perhaps causally so–for the effects of a given intervention” (p. 309). Mechanisms can either be common or unique across diverse interventions. Only a handful of articles describe potential mechanisms of change by which the effectiveness of MT could be explained. Anderson and Patel [15] used a stress regulation-based framework of how music might work in order to reduce stress in preterm babies hospitalized in the NICU. The authors part from the fact that the NICU setting can activate a stress response via the hypothalamic–pituitary–adrenal (HPA) axis in hospitalized babies, which may adversely impact neurodevelopmental outcomes by “diverting resources and energy away from brain growth, by impact on neural structures sensitive to stress hormones, and/or by impact over time through epigenetic mechanisms” (p. 264). Music may have a beneficial role “by abating the stress response, by providing a signal of positive social contact, and by offering a form of environmental enrichment” (p. 258). The authors draw the conclusion that music could be seen as “a surrogate supportive social signal” for the child isolated in the NICU [15]. Filippa and colleagues [16] described the potential of early vocal contact and music-based intervention as a “auditory sensory medium” (p. 250), which brings benefit for the preterm infant’s brain development. The authors draw their conclusions mainly in the context of neural plasticity mechanisms during sensitive and critical periods of brain plasticity and resilience, measured with magnetic resonance imaging and electroencephalography. However, the results of the review are limited to interventions implemented by trained medical staff and nurses [16]. Chorna and colleagues [17] undertook the task of describing neuroprocessing mechanisms of music on brain functioning and brain structure as well as the role of early interventions based on music and musicality in terms of early social and emotional development and environmental enrichment. Haslbeck and Bassler [18] described a neuroscience-based framework of MT for preterm infants and their parents, in which the authors acknowledge the auditory perspective in newborns as recognition and preference of the maternal voice and female voices as well as auditory brain plasticity and cortex development. According to the authors, the appropriateness of auditory stimulation through music might activate limbic and paralimbic regions promoting social contact, which are milestones in brain maturation after birth [18]. In another perspective paper, Shoemark, Hanson-Abromeit, and Stewart [19] stressed that music-based interventions optimize the experience and environment of preterm infants, especially when they are delivered by music therapists with parental involvement. The authors referred to typical environment features of NICUs (such as unpredictable noise, invasive procedures, limited social interaction), and prolonged or excessive stress in hospitalized babies, which have an impact on the faster acting sympathetic-adrenal system and the slower acting HPA axis [19].

The aim of this article is to make a first step towards the discussion of potential mechanisms in the field of neonatal MT, specifically when considering interventions aimed at parents and preterm babies during kangaroo care [20]/skin-to-skin contact (SSC). SSC is a widespread procedure improving cardio-respiratory stabilization [21], temperature regulation, stress or pain responses, behavioral states, and sleep organization during the neonatal period. SSC improves long term cognitive and psychomotor development [22].

## 2. Potential Mechanisms: Hypothalamic–Pituitary–Adrenal Axis, Autonomic Nervous System, and Co-Regulation

Following preterm delivery, the environmental situation changes significantly from the intrauterine environment to the extrauterine environment. The hospitalization and pain-related events due to medical procedures (acute procedural, and acute prolonged) expose the child to several sources of stress, such as environmental stress, as well as atypical maternal care [23]. Environmental stress is linked to noncyclical and bright light exposure, loud noises (many times exceeding the recommended sound level of 45 dB), gravitational forces, frequent handling, or cool and dry temperature. On the contrary, the intrauterine environment is characterized by a warm environment, rhythmic and cycling stimulation linked with maternal activity and hormonal cycles, flex position of the baby, uterine walls which offer boundaries, gently rocked in fluid, and low-frequency sounds [24]. Stress systems are activated when there is a discrepancy between an internal organism’s expectation and an external situation that deviates from the body’s needs, mostly when the body cannot achieve homeostasis. An allostatic response is a complex path of adaptation and coping to achieve physiological stability (homeostasis) through behavioral and/or physiological changes as a response to environmental stressors. Compared to homeostasis, allostasis is a more dynamic process, enabling the body to cope with larger changes in response to changing conditions. Weber, Harrison, and Steward [25] stressed that with respect to preterm infants, allostatic load refers to the total cost to the body due to long periods of high stress as a result of running multiple allostatic processes, which are repeated, prolonged, and not quickly nor not adequately solved. This causes adverse physiological and psychological outcomes such as neurodevelopmental delays, socioemotional problems, and poor self-regulation. The main mechanisms of allostasis are the autonomic nervous system (ANS), the HPA axis, the immune system, the cardiovascular system, and many others.

### 2.1. The Hypothalamic–Pituitary–Adrenal Axis and the Preterm Infant’s Behavioral State

The HPA axis plays a pivotal role with regard to stress responses and the preterm infant’s behavioral state. The HPA axis is a nervous part of the neuroendocrine system (consisting of several interconnected centers in the brain and body) responsible for maintaining physiological homeostasis and is therefore one of the mechanisms by which a human’s response to stress can be described. The HPA axis in preterm babies is immature and has “reduced capability of the adrenal glands to produce cortisol secondary to intermediate deficiency in the steroidogenesis pathway, but with rapid recovery if the hormonal axis in early (app. 14 days) postnatal life” [24] (pp. 86–87). Additionally, preterm infants do not produce the adequate quantity of stress hormone for the degree of stress or illness experienced [24,26], which all together contribute to their difficulty in achieving homeostasis. Research shows that there is a connection between the adrenal cortex function and gestational age, which is determined by the fact that the HPA axis matures gradually during the third trimester of gestation [27]. The delay in maturation of the HPA axis is known as transient adrenocortical insufficiency of prematurity. Among the risk factors for adrenal insufficiency are gestational age below 30 weeks, early postnatal age, perinatal stress (linked with the stressful situations and postnatal illness), and duration of corticosteroid use [24]. Lammertink and colleagues [23] suggest that the impact of prematurity provides long-lasting changes in HPA-functioning, while the timing of the stressor plays a fundamental role to the type and magnitude of the stress-responses in preterm babies, as well as that it might contribute to a differential impact and reaction (as hypo- or hyper-reactivity).

### 2.2. The Autonomic System and the Preterm Infant’s Physiologic State

The ANS is the part of the nervous system that supplies the internal organs, including the blood vessels, stomach, intestine, liver, kidneys, bladder, genitals, lungs, pupils, heart, and sweat, salivary, and digestive glands. The ANS has two main divisions: a sympathetic and a parasympathetic. After the ANS receives information from the internal environment and external environment, it responds by stimulating body processes, usually through the sympathetic division, or inhibiting them, usually through the parasympathetic division. Many organs are controlled primarily by either the sympathetic or the parasympathetic division. Sometimes the two divisions have opposite effects on the same organ. For example, the sympathetic division increases blood pressure and the parasympathetic division decreases it. Overall, the two divisions work together to ensure that the body responds appropriately to different situations. Generally, the sympathetic division prepares the body for stressful or emergency situations—“fight or flight”; thus, the sympathetic division increases heart rate and the force of heart contractions and dilates the airways to make breathing easier. It causes the body to release stored energy. It also slows body processes that are less important in emergencies, such as digestion and urination. The parasympathetic division controls body processes during ordinary situations. Generally, the parasympathetic division conserves and restores energy. It slows the heart rate and decreases blood pressure. It stimulates the digestive tract to process food and eliminates wastes. At birth, the ANS plays a major role in the successful transition from the fetal to the extra-uterine environment. The latter half of gestation and early neonatal periods are critical periods for maturation of the ANS [28]. Consequently, preterm birth has two important consequences for the developing ANS. First, autonomic maturation in the preterm infant may be underdeveloped and ill-prepared to support the profound physiological changes at birth. Second, subsequent developmental changes occur in a vastly different and ‘unnatural’ extra-uterine milieu [28]. Early disruption of the ANS development limits the infant’s ability to respond to the environment [29].

### 2.3. Co-Regulation and Bonding

Hospitalization in the NICU may lead to atypical maternal care due to limited contact with the parent (s), who should take on the role of a primary caregiver (s) at this time. This refers especially to babies who are born with a low gestational age or/and are vulnerable to developmental risks, because such conditions usually prolong their stay at the NICU. Admission at the NICU has an adverse impact on a child’s physical and emotional contact with the mother, which may lead to feelings of isolation and abandonment. At the same time, parents experience helplessness, anxiety, stress, sadness, and are being pushed out of the role of being the first caregiver which may in turn negatively impact parent-infant bonding (and later on attachment) and parental mental health [30]. The Newborn Individualized Developmental Care and Assessment Program (NIDCAP) and SSC suggest that a strong link may exist between physical and emotional closeness of a vulnerable infant and his/her parents and the child’s behavioral outcomes, feeding abilities, and physiological state. It may therefore be assumed that physical and emotional closeness between parents and preterm infants may have an indirect impact on MT during SSC and could play a moderator role in its effectiveness. Because MT along with SSC can intervene favorably in both preterm infants and their parents, the effect of the mother might be much more crucial for the baby in terms of the interaction between them than that of intervention itself. MT interventions are dedicated not only for the baby, but also for the caregiver; thus, co-regulation seems to be a key concept. On the other hand, much of the current literature on parenting behaviors pays particular attention to fostering the successful development and well-being of an infant, while in cases of prematurity, the quality of mothers’ interactional behaviors might differ from those mothers who have a full-term baby. Although research results are inconclusive, it cannot be overlooked that some studies indicate adverse preterm birth-related parental behaviors (e.g., less sensitive and more intrusive behaviors in mothers of preterm infants).

## 3. Main Results from RCTs of Music Therapy during Kangaroo Care Focusing on the Preterm Infants’ Responses and Mother-Infant Bonding

In this section we will discuss in detail the main results of MT during SSC, focusing on infant- and/or caregiver-related variables and excluding studies with designs that are less rigorous than RCTs. We particularly present the data related to preterm infants’ response in infants’ behavioral state explained by the HPA axis, related to preterm infants’ response in infants’ physiologic state explained by the ANS, and parent-infant interaction explained by co-regulation. Table 1 presents some of the main characteristics of the eligible studies along with the results.

### 3.1. Preterm Infants’ Response in Infants’ Behavioral and Physiological Outcomes

Recently, a considerable amount of literature has been published around the theme of preventing or reducing postnatal stress. However, there is a general lack of RCT-evaluated MT during SSC in terms of preterm infants’ responses to sources of stress while being at the NICU. To our best knowledge, data about the effectiveness of this dual treatment (MT and SSC) on infant behavioral outcomes are limited to two crossover RCTs conducted in Israel (Table 1). In the first study, Arnon and colleagues [31] evaluated the effect of maternal singing during KC compared to KC alone, but have not found a significant effect on the infants’ behavioral states. In the second one, Epstein and colleagues [32] also assessed the effect of maternal singing during KC compared to KC alone on infants’ behavioral states, but have found that maternal singing during KC induced behavioral instability. Despite the common characteristics of the two studies (e.g., MT approach, country of origin, and type of the study) they differ in terms of the population included. The first one [30] targeted stable preterm infants, while the second one [31] preterm infants with severe brain damage; therefore, we might conclude that the HPA axis might act differently in the heterogenous population of premature infants in the same way as the ANS act differently in these two studies.

There is a growing body of research that recognizes the importance of MT on the preterm infant’s physiological state which can be explored with physiological outcomes of the ANS. Among NICU-MT studies, researchers mostly concentrated on three physiological parameters: HR, RR, and oxygen saturation (O2 Sat). Heart rate variability (HRV) has recently become a powerful tool for studying the ANS and provides a measure of sympathetic and parasympathetic functioning and therefore ANS maturation [34,35,36]. Unlike HR, which focuses on the average beats per minute controlled by hemodynamic changes, HRV measures the specific changes in time (or variability) between successive heart beats and is controlled by the ANS. As far as we know, only two RCTs (both conducted in Israel; Table 1) have measured the effect of MT exposed during KC on HRV so far [31,32], with one showing positive effects of combined MT interventions with SSC on ANS maturation compared to SSC alone [31]. In contrast to our expectations, there is much less evidence about the effects of MT interventions in combination with KC on physiological parameters. Arnon and colleagues [31] in the aforementioned research have found that combining maternal singing with KC causes autonomic stability as indicated by the ratio of low-frequency power (indicating sympathetic power) divided by high-frequency power (indicating parasympathetic power). The lowest value of this ratio indicates better stability of the ANS, but no significant effects have been found for other physiological outcomes [31]. In a RCT pilot study conducted in Colombia, Ettenberger and colleagues [33] have compared the effect of MT using three arms: MT during KC with parents, MT with the babies alone, and a control group, looking at outcomes such as HR, O2 Sat, and growth (Table 1). A trend was found towards an increased weight gain for both intervention groups and a shorter length of hospitalization for one of the intervention groups was noticed; however, no significant effect was found regarding physiological outcomes such as O2 Sat and HR.

### 3.2. Parent-Infant Interaction

While the integration of parents to study protocols with music medicine and MT interventions has increased over the last 10 years [37], parental outcomes–and specifically those related to the parent-infant relationship–are still scarce. So far only a couple of studies have tried to measure the impact of music interventions in relation to the parent-infant relationship, either through directly measuring bonding [33,38,39,40,41,42] or indirectly through observing parent-infant interactions during play or care activities [43]. The results are inconclusive so far. Most of the beforementioned studies showed positive trends, but none managed to report statistically significant results favoring the MT groups. The lack of RCTs evaluating the parent-infant interaction impact of MT exposed during KC with premature infants and their caregivers represents a major gap in the literature. To our best knowledge only one completed study (Table 1) [33] and one ongoing study include bonding as an outcome where MT was combined with SSC [44]. In addition, the parent-infant relationship is a relatively vague concept and thus difficult to measure. While bonding has been used most commonly as the main outcome measure of the early parent-infant relationship, a clear differentiation between bonding and attachment is further hindering theoretical advances that allow the description of potential mechanisms by which MT interventions might achieve significant changes [45].

## 4. Potential Variables Related to the Mechanisms in NICU Music Therapy during Kangaroo Care

Both MT and SSC can intervene favorably in both preterm infants and their parents. At the same time, there is the interconnectedness and complexity of biological and psychological factors influenced on this dual treatment’s effectiveness. In this section we will discuss in detail the potential infant- and/or caregiver-related variables which are related to the aforementioned mechanisms.

### 4.1. Preterm Infants’ Responses Related to the HPA-Axis and ANS in Infants’ Behavioral and Physiological Outcomes

Few infant-related variables might play a pivotal role while considering the effectiveness of MT combined with KC on a child’s behavioral and physiological outcomes. We strongly believe that gestational age, postmenstrual age at the start of therapy, and the type and degree of postnatal morbidity and comorbidity are among the most important factors for the treatment’s effectiveness. Although no formal statistical analysis has been performed to prove this hypothesis, this is in line with some of the studies investigating the effectiveness of other early therapies provided for preterm infants during their NICU stay. For instance, Pratiwi et al. [46] have found that gestational age is one of the few predictors of SSC on infant weight gain, HR, RR, and body temperature regulation. The ANS maturation and behavioral responsivity in preterm infants is largely conditioned by gestational age [29], which is also a medical factor determining the starting point of many early therapy services in the NICU. In the field of NICU-MT it has been recommend that interventions should not be started earlier than 26 weeks [47].

Presence and degree of postnatal morbidity and comorbidity could also play a fundamental role in the effectiveness of MT interventions combined with KC. Postnatal complications are directly linked with the child’s clinical condition. From a practical point of view, it seems that a greater role should be attached to the child’s medical stability than to the gestational age, due to the fact that late preterm infants (born between 34 and 36 weeks 6 days of gestation) are also at risk for clinical problems in the postpartum period even they are born only a few weeks early [48]. A recent RCT by Epstein and colleagues [32] measuring the effect of maternal singing combined with KC on 35 preterm infants with severe brain damage has shown interesting results. The authors have found that maternal singing during KC induced physiological and behavioral instability and increased maternal anxiety during NICU hospitalization. Epstein and colleagues [32] suggested that maternal anxiety levels as well as infants’ music processing abilities were impacted by the infants’ brain injury, leading to the ANS instability. This study was the first MT study with preterm infants with brain damage (intraventricular hemorrhage (IVH) grades 3–4 and Periventricular Leukomalacia (PVL) (levels 3–4), a population usually excluded from studies. In a previous study [31] healthy preterm infants and preterm infants with mild brain injury (IVH grade 1–2) were included and received the exact same intervention (maternal singing during SSC compared to SSC alone), leading to positive results on ANS stability. The discrepancy between the findings of the two aforementioned studies [31,32] may serve as grounds to suggest potential infant-related variables (specifically brain injury) as a possible factor of MT effectiveness. While it has been shown that SSC on its own supports the ANS [22], and combined SSC with MT increases ANS stability on healthy preterm infants [31], these new findings [32] suggest that maternal anxiety as well as infants’ music processing abilities might be central mediators for ANS functioning. We further suggest that when MT combined with SSC is offered to preterm infants with no brain damage, maternal anxiety levels are reduced [31], activating a co-regulation sub-mechanism through which the infant is able to respond to the parent’s decreased levels of stress in a similar matter, leading to an improvement in the infants’ ANS stability. Additionally, when infants’ music processing capabilities are not disturbed by severe brain injury, the infant is more able to process the music delivered by the parents during the intervention, turning it to a positive stimulation also supporting ANS stability.

### 4.2. Parent-Infant Interaction Relaetd to Co-Regulation in Bonding

Human relationship formation is unquestionably a complex phenomenon, and the early parent-infant relationship is no exception. In the NICU, several studies have highlighted the risk that preterm birth and the subsequent hospitalization may pose to the developing bond between parents and their baby [49,50,51,52]. Few variables might play a pivotal role while considering the effectiveness of MT combined with KC on early parent-infant interaction, e.g., breastfeeding, maternal mental health, maternal sensitivity, and interactional synchronicity. For instance, Ettenberger et al. [40] reported that parents with higher bonding scores at baseline seemed to benefit more from MT compared to parents with low baseline scores. Although no formal statistical analysis has been performed to prove this hypothesis, this is in line with some of the studies on other early parenting interventions, which showed that higher depression levels led to improved treatment. In relation to MT during SSC, the situation is more complex, since SSC itself moderates parental mental health (i.e., anxiety levels) through oxytocin emission and inhibition of stress biomarkers such as cortisol [53]. Other studies and meta-analysis showed that early SSC positively impacts breastfeeding postpartum [54,55] and breastfeeding impacts again both maternal mood, affect, and stress, as well as child development [56]. Although not measuring the early parent-infant relationship, a previous study with mothers of preterm babies demonstrated a statistically significant increase in breastfeeding in mother who participated in MT sessions compared to control mothers [57]. Thus, when considering potential factors related to MT in the NICU and the early parent-infant relationship, both SSC and breastfeeding should be taken into account.

Evidence suggests that maternal mental health is among the most important factors for an early parenting intervention program’s effectiveness. Gardner and colleagues [58] found that male and younger children, and children of mothers with higher depression levels at baseline, benefited more from the intervention resulting in less conduct problems. Socio-demographic factors such as income did not moderate the results in this case. On the other hand, the authors found that fostering positive parenting rather than reducing negative parenting mediates child conduct problems [58]. Another study by Adam and colleagues [59] measured the relation of the mothers’ own attachment representations identified with the Adult Attachment Interview (AAI) with maternal wellbeing and parenting behavior. Dismissing or preoccupied mothers showed lower levels of positive affectivity and higher levels of negative affectivity, anxiety, or intrusive parenting behaviors. Especially in mothers with elevated depression levels, dismissing attachment was related to lower warmth/responsiveness in parenting. Further, two other studies including Polish mothers and fathers of full-term babies showed that both postpartum depression and maternal stress are correlated with mother-infant bonding after birth [60], but paternal bonding was only affected by high levels of stress in the participating fathers [61].

In a meta-analysis on parental antecedents and infant attachment De Wolff and colleagues [62] described five clusters of parent-infant interaction, including synchrony, mutuality, positive attitude, emotional support, and stimulation. The authors concluded that “Maternal sensitivity, defined as the ability to respond appropriately and promptly to the signals of the infant, indeed appears to be an important condition for the development of attachment security” and “…that is, infants whose mothers respond sensitively to their signals improve their chance of developing a secure relationship from 38% to 62%, whereas infants whose mothers are less sensitive decrease their chance of developing a secure relationship from 62% to 38%” [62] (p. 584). In the context of NICU-MT the formation of interactional synchronicity between a parent and a child is a variable by which children feel calmer, safe, and loved, which is extremely important when children are hospitalized. Developmental psychologists say that interaction synchrony is an innate ability based on physiological mechanisms (oscillator systems, and attachment-related hormones) and ”addresses the matching of behavior, affective states, and biological rhythms between parent and child that together form a single relational unit” [63] (p. 329). In the context of prematurity, a recent study confirmed that both prolonged daytime sleep and positive parenting (i.e., affect, responsiveness, sensitivity, etc.) of mothers of preterm babies at 9 months were associated with higher levels of secure attachment at 16 months [64]. A third study showed that maternal self-representation was a key predictor for post-partum attachment (PPA), besides environmental stressor sensitivity. Both environmental stressor sensitivity and the mothers’ perception of the quality of family-centered care influenced indirectly PPA through psycho-emotional responses in mothers, such as mood and posttraumatic stress [65]. Specifically for at-risk babies, one study found parent-infant interaction to be a mediator for cognitive development at 12 months, but similarly to previous studies, sociodemographic variables of the mothers did not moderate the relation between neonatal risk and cognitive development [66].

In conclusion, there seems to be evidence that parental mental health, parental sensitivity, and parent-infant interaction are important domains for developing a secure and stable parent-infant relationship. This is highly relevant since it is known that parental mental health can be affected by preterm birth and the NICU hospitalization [67]. While parental participation in both MT clinical practice and research is on the rise, specific focus on bonding and attachment and parental mental health has been stressed by various authors [41,42,68,69,70]. On the other hand, MT consciously tries to foster positive parenting by providing a safe space for parents to creatively interact with their infants, and music therapy as part of family-centered care can improve the cognitive appraisal of parents and indirectly affect attachment. Susceptibility to treatment will certainly be an important part of future research.

## 5. Implication for Further Research

Due to the difficulties in testing mechanisms as such, researchers usually take into account mediators and moderators as a method of indicating treatment mechanisms [14]. In treatment research a moderator aims to describe for whom or under what conditions MT would work and refers to a characteristic of the patient, therapist, or treatment delivery. The potential role of mediators, on the other hand, turns the thinking of results from ‘effect of a cause’, to the ‘cause of the effect’ [13]. From the statistical point of view, a mediator is an intermediate variable which shows statistical relations between intervention and outcomes of interest, while a moderator refers to the direction or magnitude of the association between intervention and outcomes of interest [13]. Identifying moderators or mediators in MT research is a rather new phenomena but has been mentioned as an important aspect for future research in medical settings [71]. In a recent meta-analysis of MT and psychosocial treatment in adult cancer patients, for example [72], the authors identified single sessions and receptive techniques as moderators for psychosocial wellbeing. In another meta-analysis, study type (i.e., quasi-experimental studies), origin of study (i.e., non-western countries), type of control condition (i.e., waiting list design), or study quality (i.e., low quality studies) were found to positively impact physiological and psychological stress-related outcomes via MT treatment [73]. To our best knowledge, a formal moderator-mediator analysis focusing on the parent-infant relationship and preterm infants’ responses for MT during KC in the NICU has not yet been performed. In the body of this manuscript, we have discussed potential infant and/or caregiver variables related to this dual treatment; however, several other issues remain unanswered at present.

To develop a full picture of variables related to MT exposed during KC in the NICU we propose the following domains for future research:

1. Patient-related variables for preterm infants (e.g., gestational age, postmenstrual age at the start of therapy, birth weight, the type and degree of postnatal morbidity and comorbidity, and diagnosis).
2. Patient-related variables for parents/caregivers of preterm infants (e.g., mental health; levels of stress or anxiety; presence of depressive symptoms; personality traits; parental self-efficacy, sensitivity, and attunement; parental competences; and being a first time-parent).
3. Therapist-related variables (e.g., age, gender, experience, and profession: music therapists vs. other health care professionals).
4. Intervention related variables (e.g., dose, duration, frequency, timing, mode of referral: self vs. healthcare provider, and mode of therapy: in-person vs. online).
5. MT approach related variables (e.g., with family or baby alone, type of MT approach, type of delivery: earphones vs. free field vs. live music, and active participation of parents vs. listening).

From our perspective, it is important to move beyond evidence-based research to explore how NICU-MT might work, why NICU-MT works, what approach would bring the most benefits, or for whom and under what conditions NICU-MT works best. Such questions represent what Guralnick [74] refers to as ‘second-generation research’–namely “questions that examine the mechanisms through which interventions produce their benefit and attempt to identify which specific interventions will work best for specific individuals” [75] (p. 2).

## 6. Conclusions

In this perspective article we discussed a few potential mechanisms of MT interventions, specifically considering interventions aiming at parents and preterm babies during KC or SSC. Furthermore, the article provided insights into potential infant- and/or caregiver-related variables, which are related to the HPA axis, the ANS, and parent-infant co-regulation; and therefore, to a possible intervention course in future NICU research. Using MT interventions dedicated to preterm babies during their hospitalization is supported by the best empirical data for several outcomes [7,8,9,10,11,12], but findings of MT exposed during KC on the preterm infant’s response (behavioral and physiological outcomes) and mother-infant interaction provide only some support for the conceptual premise on its effectiveness [31,32,33,39]. Since a formal moderator or mediator analysis has not yet been performed for research of MT combined with KC during the babies’ stay in the NICU, the mechanisms discussed in this article are hypothetical ones and are drawn from knowledge of other related fields, such as medicine, psychology, psychiatry, and psychotherapy. We hope, however, that this discussion incentivizes future research to start considering moderators and mediators as part of their data analysis in order to help generate new knowledge regarding treatment susceptibility and optimizing resources. Thus, the discussion of potential mechanism and factors related to its implementation would provide input for improvement of currently available MT approaches. In addition, knowledge about mechanisms might contribute to further understanding variables related to the course of prematurity and other early interventions provided in the NICU.

## Figures and Tables

**Table 1:** Study characteristics on music therapy during kangaroo care focusing on the preterm infants’ responses and parent-infant interaction.

References	Country	Design	N of Participant Included	Number of Groups	Intervention Group Description	Preterm Infant’s Behavioral State (*p*-Value)	Preterm Infant’s Physiologic State (*p*-Value)	Parent-Infant Interaction (*p*-Value)	Other Outcomes (*p*-Value)
Arnon, 2014 [31]	Israel	crossover RCT	86 mother–infant dyads	MT during KC KC	2 sessions of live maternal singing for 40 min over 2 days; [10 min of KC alone, followed by KC and MT for 20 min; and 10 min of KC alone]	behavioral states * (*p* = 0.7)	HR (*p* = 0.67), RR (*p* = 0.38), O2 Sat (*p* = 0.23) HRV (LF/HF ratio, *p* = 0.04)		maternal anxiety ** (*p* = 0.03), mother’s HR (*p* = 0.21), mother’s RR (*p* = 0.31), mother’s O2 Sat (*p* = 0.18)
Epstein, 2021 [32]	Israel	crossover RCT	35 infants	MT during KC KC	3 sessions of live maternal singing for 40 min over 3 days; [10 min of KC alone, followed by KC and MT for 20 min; and 10 min of KC alone]	behavioral states *** (*p* = 0.03)	HR (*p* = 0.04), RR (*p* = 0.04), O2 Sat (*p* = 0.04) HRV (LF/HF ratio, *p* = 0.01)		maternal anxiety ** (*p* = 0.04); mother’s HRV (*p* = 0.07); mother’s O2 Sat (*p* = 0.15);
Ettenberger, 2014 [33]	Colombia	parallel RCT	30 infants; 26 parents	MT with parents during KC; MT with the babies alone Standard care	4 sessions of MT interventions for 8–25 min over 14 days Group 1: singing songs with the parents (lullabies/children’s songs/other songs); Group 2: Interventions with the use of the accompanying instruments (guitar, ocean drum, gato-box, ocean drum alone, mixed instruments).		HR (n/a) O2 Sat (n/a), weight gain (*p* = 0.05)	Bonding **** (*p* = 0.49)	maternal anxiety ** (*p* = 0.01 for Factor 2 before the first intervention; *p* = 0.0418 for Factor 6 after the last intervention), Length of Hospitalization (*p* = 0.39)

Annotation: HR, heart rate; HRV, heart rate variability; LF/HF, low frequency/high frequency; n/a, not available; O2 Sat, oxygen saturation; *p*, significant; RCT, randomized controlled trial; RR, respiratory rate. Notes: * based on a seven-point score based on the criteria of Als to assess the infants’ behavioral states as: deep sleep, light sleep, drowsy, quiet awake or alert, actively awake and aroused, highly aroused, upset or crying and prolonged respiratory pause of more than eight-seconds; ** STAI, State-trait anxiety inventory; *** NIDCAP manual for naturalistic observation and the Brazelton Neonatal Behavioral Assessment; **** MIBS, other-to-Infant Bonding Scale.
